# Insulin-like growth factor-I gene delivery to astrocytes reduces their inflammatory response to lipopolysaccharide

**DOI:** 10.1186/1742-2094-8-21

**Published:** 2011-03-03

**Authors:** Maria J Bellini, Claudia B Hereñú, Rodolfo G Goya, Luis M Garcia-Segura

**Affiliations:** 1 Instituto Cajal, CSIC, Madrid, Spain; 2 INIBIOLP-Histology B, School of Medicine, Faculty of Medicine, University of La Plata, CC 455, 1900, La Plata, Argentina

## Abstract

**Background:**

Insulin-like growth factor-I (IGF-I) exerts neuroprotective actions in the central nervous system that are mediated at least in part by control of activation of astrocytes. In this study we have assessed the efficacy of exogenous IGF-I and IGF-I gene therapy in reducing the inflammatory response of astrocytes from cerebral cortex.

**Methods:**

An adenoviral vector harboring the rat IGF-I gene and a control adenoviral vector harboring a hybrid gene encoding the herpes simplex virus type 1 thymidine kinase fused to *Aequorea victoria *enhanced green fluorescent protein were used in this study. Primary astrocytes from mice cerebral cortex were incubated for 24 h or 72 h with vehicle, IGF-I, the IGF-I adenoviral vector, or control vector; and exposed to bacterial lipopolysaccharide to induce an inflammatory response. IGF-I levels were measured by radioimmunoassay. Levels of interleukin 6, tumor necrosis factor-α, interleukin-1β and toll-like receptor 4 mRNA were assessed by quantitative real-time polymerase chain reaction. Levels of IGF-I receptor and IGF binding proteins 2 and 3 were assessed by western blotting. The subcellular distribution of nuclear factor κB (p65) was assessed by immunocytochemistry. Statistical significance was assessed by one way analysis of variance followed by the Bonferroni pot hoc test.

**Results:**

IGF-I gene therapy increased IGF-I levels without affecting IGF-I receptors or IGF binding proteins. Exogenous IGF-I, and IGF-I gene therapy, decreased expression of toll-like receptor 4 and counteracted the lipopolysaccharide-induced inflammatory response of astrocytes. In addition, IGF-I gene therapy decreased lipopolysaccharide-induced translocation of nuclear factor κB (p65) to the cell nucleus.

**Conclusion:**

These findings demonstrate efficacy of exogenous IGF-I and of IGF-I gene therapy in reducing the inflammatory response of astrocytes. IGF-I gene therapy may represent a new approach to reduce inflammatory reactions in glial cells.

## Background

As a source of growth factors and of immunologically relevant cytokines and chemokines, astrocytes play a pivotal role in the pathophysiology of neurodegenerative diseases [1-3] and in the type and extent of central nervous system immune and inflammatory responses [4]. These responses may promote tissue repair and contribute to recovery of homeostasis under acute neurodegerative conditions. However, sustained inflammatory responses of astrocytes in chronic neurodegenerative diseases may enhance tissue damage through amplification of brain inflammation and consequent neuronal injury [4-8]. Therefore, to limit neuronal cell death under chronic neurodegenerative conditions, it is important to develop tools to control brain inflammatory reactions.

IGF-I is locally produced in the nervous system and it is also actively transported to the brain from plasma through the choroid plexus [9,10]. IGF-I has pleiotropic actions in nervous tissue, influencing neuronal development, synaptic plasticity, neuroendocrine regulation, adult neurogenesis and cognition [11-14]. IGF-I is also a potent neuroprotective molecule [10,13,15,16] exerting this function in part by reducing brain inflammation [17,18] and reactive astrocytosis [19]. In response to neurodegenerative conditions, astrocytes express IGF-I, probably as an endogenous neuroprotective and anti-inflammatory mechanism [20-23]. Consequently, the development of methodologies to increase IGF-I production by glial cells is a logical approach to implement anti-inflammatory IGF-I-based therapeutic strategies for neurodegenerative diseases.

We have recently constructed a recombinant adenovirus vector harboring the rat IGF-I gene [24,25]. Using this vector we have shown efficacy of IGF-I gene therapy to increase IGF-I levels in cerebrospinal fluid and to reduce neuronal damage in vivo [24,25]. In the present study we have explored whether exogenous IGF-I and IGF-I gene therapy regulate the inflammatory response of astrocytes.

## Methods

### Adenoviral vectors

**Figure 1:**
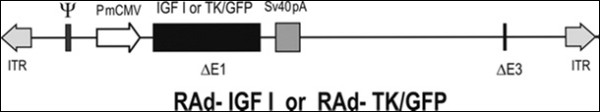
**Schematic representation of the construction of the RAd-IGF-I and RAd-TK/GFP adenoviral vectors**. PmCMV, mouse cytomegalovirus promoter; IGF-I, cDNA for rat IGF-1; TK/GFP, hybrid DNA sequence encoding the fusion protein TK/GFP; ITR, inverted terminal repeat; ΔE1 and ΔE3, deletions in the AD5 genome; SV40, simian virus 40 polyadenylation signal; Ψ, packaging signal.

A recombinant adenovirus (RAd) vector harboring the rat IGF-I gene (RAd-IGF-I) was constructed as previously described [24] using a variant of the two-plasmid method [26] The cDNA coding for the rat IGF-I gene (kindly donated by Dr. Peter Rotwein, Department of Biochemistry and Molecular Biology, Oregon Health & Science University, Portland, OR), obtained from the mRNA for the IGF-Ib precursor form [27], was placed under the control of the mCMV promoter in order to construct the genome of the desired RAd-IGF-I (Figure 1). The newly generated RAd was rescued from human embryo kidney 293 (HEK293) cell lysates and plaque purified. It was further purified by ultracentrifugation in a CsCl gradient. Final virus stocks were titrated by a serial dilution plaque assay.

A control RAd vector (RAd-TK/GFP) harboring a hybrid gene encoding the herpes simplex virus type 1 (HSV-1) thymidine kinase fused to *Aequorea victoria *enhanced green fluorescent protein (a kind gift from Dr. Jacques Galipeau, McGill University, Montreal, Canada) was constructed following the general procedures outlined above (Figure 1). The corresponding gene product, fusion protein TK/GFP, emits green fluorescence with high intensity when excited with 470-nm wideband light [28]. This hybrid gene is also driven by the mCMV promoter. The vector was expanded in 293 cells and purified and titrated as indicated above.

### Astrocyte cultures

Astrocyte cultures were prepared by mechanical dissociation of cerebral cortex from newborn CD1 mice [29]. Experimental procedures were approved by our Institutional Animal Use and Care Committee (Spanish National Research Council Animal Experimentation Committee). Cell culture reagents were purchased from Invitrogen (Paisley, UK). The cortex was isolated under a dissecting microscope and cleaned of choroid plexus and meninges. Cell suspensions were filtered through a 70-μm nylon cell strainer into phenol red free Dulbecco's modified Eagle medium (DMEM) containing 10% fetal calf serum and 1% penicillin-streptomycin. After centrifugation, cells were cultured in 75-cm^2 ^tissue culture flasks at 37°C and 5% CO2. The medium was changed after 4 days in culture and subsequently two times a week for the entire culture period. Cellular confluence was observed 10 days after plating, producing around 4.3 × 10^6 ^cells per flask, showing a polygonal flat morphology. Enriched astrocyte cultures (90-95% of GFAP immunoreactive cells) were obtained after overnight shaking at 37°C a 280 rpm in a table top shaker (Thermo Forma, Marietta, OH) to minimize oligodendrocyte and microglia contamination. Astrocytes were removed from the flasks by incubation with 0.25% trypsin (type II-S; Sigma-Aldrich, St Louis, MO) and 0.04% EDTA (Sigma) and plated onto poly-L-lysine-coated six-well plates or coverslips in medium supplemented with 10% FBS. Cells were plated at 15,000 cells/cm^2 ^for immunocytochemistry and at 30,000 cells/cm^2 ^for gene expression or protein assays.

### Cell treatments

One day after seeding, culture medium was changed using serum free medium and the cells were treated for 24 or 72 h with vehicle or IGF-I (50 or 100 nM), and in a second experiment the cells were treated for 24 or 72 h with RAd-IGF-I, RAd-TK/GFP or medium alone (uninfected control cells). The efficacy of infection, assessed with the RAd-TK/GFP vector, was approximately 60% of the cells in the culture. Some cultures were incubated with lipopolysaccharide (LPS, from Escherichia coli 026:B6, Sigma; 500 ng/ml) to induce an inflammatory response [30]. LPS was added for 1 h to determine NFκB subcellular localization and for 5 h to assess IGF-I receptor and IGF binding proteins by western blotting, and interleukin 6 (IL6), tumor necrosis factor-α (TNF-α), interleukin-1β (IL-1β) and toll-like receptor 4 (TLR4) mRNA levels by quantitative real-time polymerase chain reaction. In some experiments, astrocytes were incubated with the IGF-I receptor tyrosine kinase inhibitor picropodophyllin (PPP, Calbiochem, 100 ng/ml) or vehicle.

### IGF-I assay

IGF-I was extracted from supernatants by acid-ethanol cryoprecipitation after incubation for 24 h with RAd-TK/GFP, RAd-IGF-I or medium alone. IGF-I levels were assessed by radioimmunoassay as previously described [24] using antibody UB2-495 (distributed by A. F. Parlow, NHPP, NIDDK). Recombinant human IGF-I (Cell Sciences Inc., Canton, MA, USA) was used as tracer and unlabeled ligand.

### Analysis of gene expression by quantitative real-time polymerase chain reaction (q-PCR)

Interleukin 6 (IL6), tumor necrosis factor-α (TNF-α), interleukin-1β (IL-1β) and toll-like receptor 4 (TLR4) mRNA levels were assessed by quantitative real-time polymerase chain reaction. Cells were lysed and total RNA was extracted using an illustra RNAspin Mini RNA Isolation Kit (GE Healthcare, Buckinghamshire, UK). First strand cDNA was prepared from RNA using an RevertAidTM H Minus First Strand cDNA Synthesis Kit (MBI Fermentas, Bath, UK) following the manufacturer's instructions. After reverse transcription (RT), the cDNA was diluted 1:4 and 5 μl were amplified by real-time PCR in 20 μl using SYBR Green master mix or TaqMan Universal PCR Master Mix (Applied Biosystems, AB, Foster City, CA) in a ABI Prism 7500 Sequence Detector (AB), with conventional AB cycling parameters (40 cycles of 95°C, 15 s; 60°C, 1 min). Primer sequences were designed using Primer Express (AB) and were as follows: for IL6, forward, 5'-GAAACCGCTATGAAGTTCCTCTCTG-3' and reverse, 5'-TGTTGGGAGTGGTATCCTCTGTGA-3'; for TNF-α, forward 5'-GAAAAGCAAGCAGCCAACCA-3' and reverse, 5'-CGGATCATGCTTTCTGTGCTC-3'; for IL-1β, forward 5'- CGACAAAATACCTGTGGCCT-3' and reverse, 5'-TTCTTTGGGTATTGCTTGGG-3'; and for TLR4, forward 5'-GGCTCCTGGCTAGGACTCTGA-3' and reverse, 5'-TCTGATCCATGCATTGGTAGGT-3'. Glyceraldehyde-3-phosphate dehydrogenase (GAPDH) was selected as control housekeeping gene. GADPH TaqMan probes and primers were the Assay-on-Demand gene expression products (AB). After amplification, a denaturing curve was performed to ensure the presence of unique amplification products. All reactions were performed in triplicate. IL6, TNF-α, IL-1β and TLR4 gene expressions were normalized to GAPDH.

### Western blotting

For western blotting analysis, medium and the cells were homogenized in 200 μl of lysis buffer (150 mM NaCl, 20 mM Tris-HCl, pH 7.4, 1% Nonidet P-40, 1 mg/ml aprotinin, 1 mg/ml leupeptin, and 1 mg/ml phenylmethylsulfonyl fluoride). Protein concentrations were determined using a Bio-Rad protein assay (Bio-Rad Laboratories, Hercules, CA). Electrophoresis of 25-μg protein extracts was performed on a 10% acrylamide SDS-PAGE gel and immunoblotted onto nitrocelulose membranes. Membranes were incubated for 1 h in TBST (10 mM Tris-HCl pH 7.6, 150 mM NaCl, 0.1% Tween-20) containing 5% wt/vol nonfat dry milk, and then incubated overnight with the primary antibodies. The following antibodies were used: anti-insulin-like growth factor-binding protein-2 (IGFBP-2; Millipore Iberica, Madrid, Spain; diluted 1/2000), anti-IGFBP-3 (H-98; Santa Cruz Biotechnology, Santa Cruz, CA; diluted 1/1000) and anti-IGF-I receptor-β (IGF-IRβ; C-20, Santa Cruz Biotechnology, Santa Cruz, CA; 1/1000). Immunoreactivity was detected with horseradish peroxidase-conjugated goat anti-rabbit or anti-mouse antibodies (Jackson ImmunoResearch Europe, Newmarket, Suffolk, UK; Diluted 1:10000) and enhanced chemiluminescence (Amersham Pharmacia Biotech, Essex, UK). Band intensities were quantified using Quantity One 1-D Analysis Software (Bio-Rad Laboratories, Inc., Hercules, CA).

### Immunocytochemistry

Subcellular distribution of NFκB (p65) was assessed by immunocytochemistry after incubation for 24 h with RAd-IGF-I or RAd-TK/GFP and for 1 h with LPS or vehicle. Cells were fixed for 15 min at room temperature in 4% paraformaldehyde, permeabilized for 15 min with 0.3% Triton X-100 in PBS and incubated for 15 min with 10% normal goat serum in PBS, to block unspecific binding of the secondary antibody. Cells were then incubated for 1 h with anti-NFκB (p65) mouse monoclonal antibody (BD Biosciences, diluted 1:50 in PBS with 1% BSA). After washing in PBS, cells were incubated with goat anti-mouse Alexa-fluor 594 (Invitrogen, diluted 1:1,000). Cell nuclei were stained with DAPI.

### Statistical analysis

Statistical significance was assessed by one-way or two-way analysis of variance (ANOVA) followed by the Bonferroni post hoc test using GraphPad Prism 5 (GraphPad Software, San Diego, CA) or by Student's t-test when only two groups were compared. A probability of P < 0.05 was adopted for statistical significance. Data shown in the figures are the results of four independent experiments. Data are represented as mean ± SEM.

## Results

### IGF-I increases the expression of IL6, IL-1β and TNF-α and decreases the expression of toll-like receptor 4 (TLR4) in astrocytes under basal conditions

**Figure 2:**
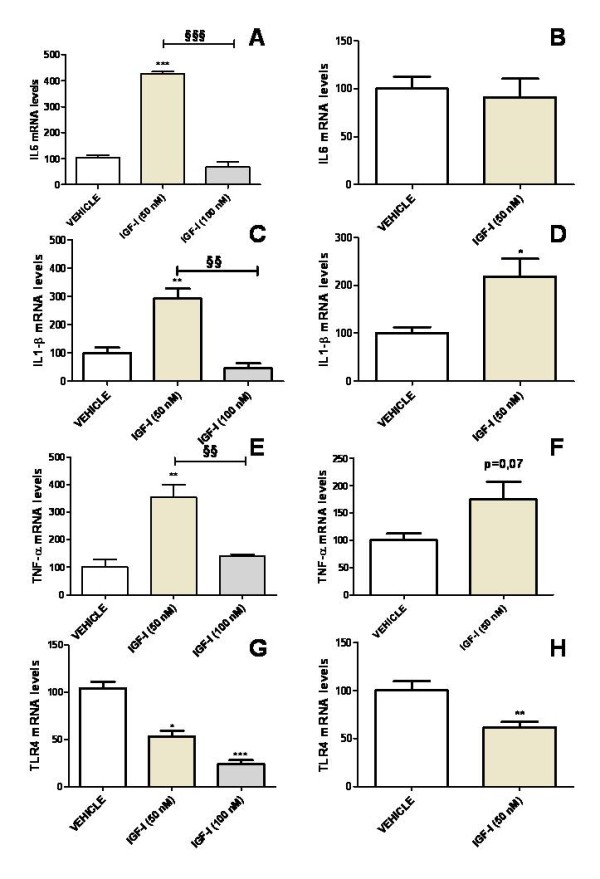
**IGF-I increases the expression of IL6, IL-1β and TNF-α and decreases the expression of TLR4 in astrocytes under basal conditions**. mRNA levels of IL6 (A,B), IL-1β (C,D), TNF-α (E,F) and TLR4 (G,H) in astrocytes 24 h (A,C,E,G) or 72 h (B,D,F,H) after the addition of IGF-I (50 or 100 nM) or vehicle to the cultures. Data are presented as mean ± SEM and are expressed as percentage of control (vehicle) values. *,**,***, Significant differences (* P < 0.05; **P < 0.01; ***P < 0.001) in comparison to the values of cultures treated with vehicle only. ^**§§**^, ^**§§§**^, Significant differences (^**§§**^P < 0.01; ^**§§§**^P < 0.001) between 50 nM and 100 nM IGF-I.

mRNA levels of IL6, IL-1β and TNF-α were assessed in astrocytes 24 h after the addition of IGF-I (50 and 100 nM) to the cultures. IGF-I, at a concentration of 50 nM, resulted in a significant increase in mRNA levels for IL6, IL-1β and TNF-α compared to astrocytes treated only with vehicle (Figure 2). In contrast, 100 nM IGF-I did not significantly affect IL6, IL-1β and TNF-α mRNA levels (Figure 2). The increase in IL-1β mRNA levels was still detected 72 h after addition of 50 nM IGF-I to the cultures (Figure 2). However, levels of IL6 and TNF-α were not significantly different between astrocytes treated with IGF-I or vehicle, 72 h after addition of IGF-I (Figure 2).

TLR4 mRNA levels were significantly decreased in cultures treated with IGF-I at either 50 nM or 100 nM, compared to cultures treated with vehicle. The effect of IGF-I on TLR4 levels was still detected 72 h after treatment (Figure 2).

### IGF-I exerts an anti-inflammatory action on LPS-treated astrocytes

**Figure 3:**
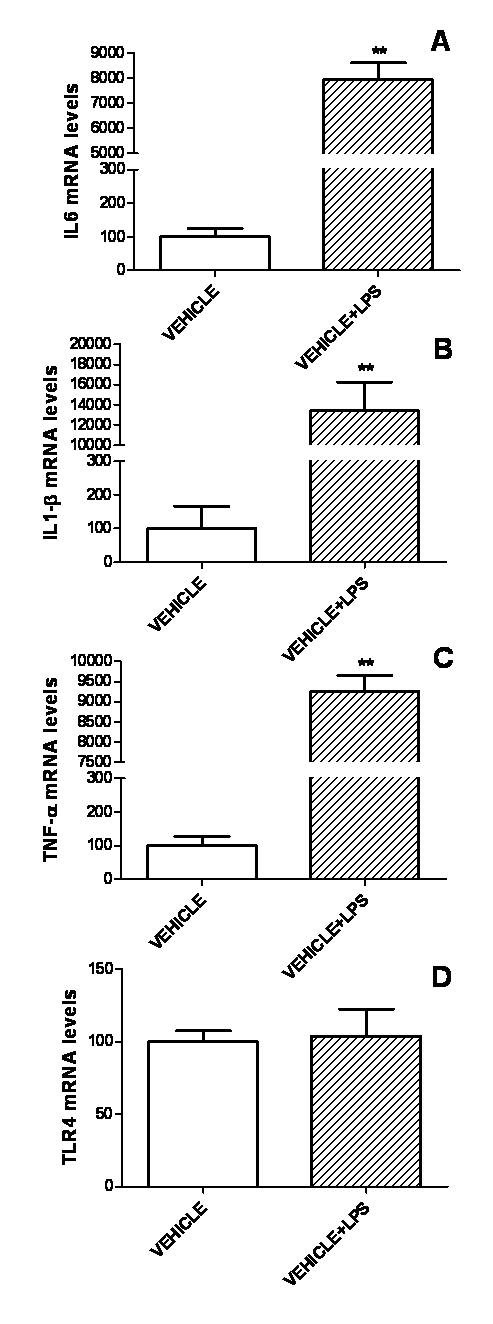
**LPS increases expression of IL6, IL-1β, TNF-α and TLR4 in astrocytes**. mRNA levels of IL6 (A), IL-1β (B), TNF-α (C) and TLR4 (D) in astrocytes treated for 5 h with LPS (500 ng/ml) or vehicle only. Data are presented as mean ± SEM and are expressed as percentage of control (vehicle) values. **,***, Significant differences (**P < 0.01; ***P < 0.001) in comparison to the values of cultures treated with vehicle.

To determine whether IGF-I affects the response of astrocytes to a proinflammatory challenge, we first assessed mRNA levels of IL6, TNF-α, IL-1β and TLR4 in primary astrocyte cultures incubated with LPS (500 ng/ml) or vehicle. Treatment with LPS resulted in a marked increase in mRNA levels of IL6, IL-1β and TNF-α (Figure 3). In contrast, TLR4 mRNA levels were not affected by LPS (Figure 3).

**Figure 4:**
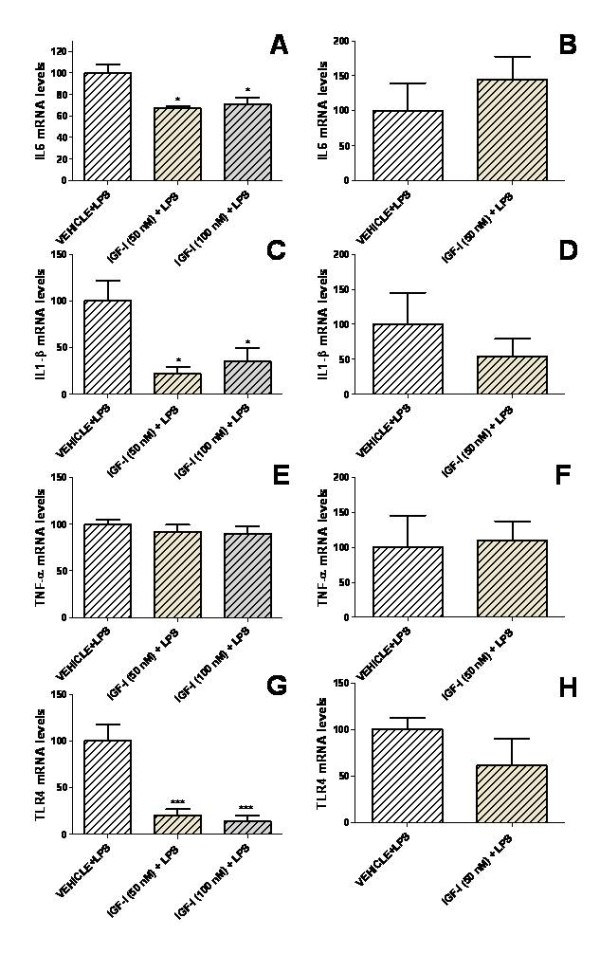
**IGF-I decreases the inflammatory response of astrocytes exposed to LPS**. mRNA levels of IL6 (A,B), IL-1β (C,D), TNF-α (E,F) and TLR4 (G,H) in astrocytes 24 h (A,C,E,G) or 72 h (B,D,F,H) after the addition of IGF-I (50 or 100 nM) or vehicle only and treated for the last 5 h with LPS. Data are presented as mean ± SEM and are expressed as percentage of control (vehicle + LPS) values. *,***, Significant differences (* P < 0.05; ***P < 0.001) in comparison to the values of cultures treated with vehicle + LPS.

For LPS-treated astrocytes, mRNA levels of IL6, IL-1β and TLR4 were significantly decreased 24 h after administration of 50 or 100 nM IGF-I in comparison to cultures treated with LPS alone (Figure 4). mRNA levels of IL6, IL-1β and TLR4 returned to control levels 72 h after addition of 50 nM IGF-I to the cultures (Figure 4).

### RAd-IGF-I increases IGF-I levels in astrocyte cultures

**Figure 5:**
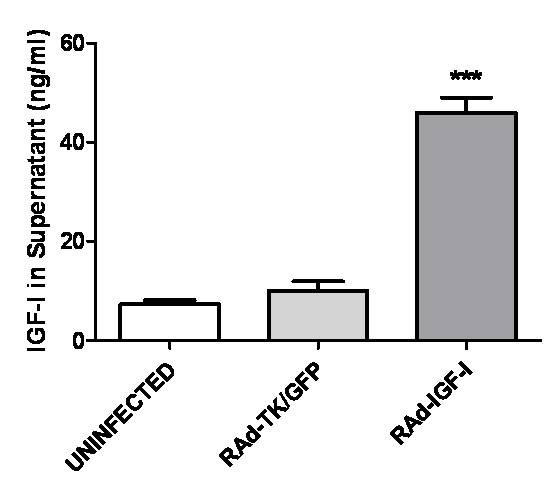
**RAd-IGF-I increases release of IGF-I by astrocytes**. Levels of IGF-I in the culture medium of astrocytes incubated for 24 h with medium alone (uninfected cells), RAd-TK/GFP or RAd-IGF-I. Total IGF-I was assayed in supernatants and the peptide concentration referred to the original volume of medium per well. Bars on columns represent mean ± SEM, (n = 4). ***, Significant difference (P < 0.001) versus the values of the other two experimental groups.

Having established the effects of exogenous application of IGF-I to astrocytes on IL6, IL-1β, TNF-α and TLR4 mRNA levels under basal and LPS-stimulated conditions, we investigated whether similar effects could be obtained by increasing endogenous expression of IGF-I in these cells using an adenoviral vector. To assess the efficacy of IGF-I gene transfer, IGF-I levels were assessed in the culture medium after incubation of the astrocytes for 24 h with RAd-IGF-I, RAd-TK/GFP, or medium alone (uninfected cells; Figure 5). IGF-I levels were significantly higher in the medium of astrocytes incubated with RAd-IGF-I compared to that of astrocytes incubated with RAd-TK/GFP or with medium alone (Figure 5). In contrast IGF-I receptor levels and IGFBP2 levels were not significantly different in astrocytes incubated for 24 h with RAd-IGF-I, RAd-TK/GFP, or medium alone (data not shown). IGFBP3 was undetectable in the cultures.

### RAd-IGF-I increases expression of IL6 and IL-1β and decreases expression of TLR4 under basal conditions

**Figure 6:**
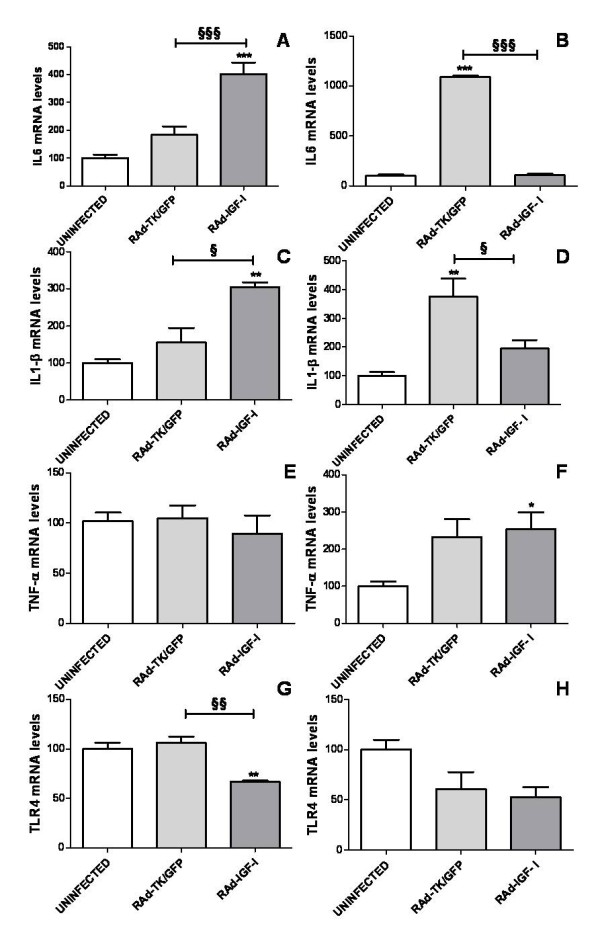
**RAd-IGF-I increases the expression of IL6 and IL-1β and decreases the expression of TLR4 in astrocytes under basal conditions**. mRNA levels of IL6 (A,B), IL-1β (C,D), TNF-α (E,F) and TLR4 (G,H) in astrocytes 24 h (A,C,E,G) or 72 h (B,D,F,H) after the addition of RAd-TK/GFP or RAd-IGF-I to the cultures. Data are presented as mean ± SEM and are expressed as percentage of control (uninfected cells) values. *,**,***, Significant differences (* P < 0.05; **P < 0.01; ***P < 0.001) in comparison to the values of uninfected cells. ^**§**^, ^**§§**^, ^**§§§**^, Significant differences (^**§**^P < 0.05; ^**§§**^P < 0.01***; ^**§§§**^P < 0.001) between RAd-TK/GFP and RAd-IGF-I.

IL6 and IL-1β mRNA levels in astrocytes were significantly increased 24 h after incubation of cultures with RAd-IGF-I, compared to those of uninfected astrocytes or to astrocytes treated with RAd-TK/GFP (Figure 6). In contrast, 72 h after incubation of the cultures with RAd-TK/GFP, astrocytes showed a significant increase in mRNA levels of IL6 and IL-1β, compared to uninfected astrocytes or to astrocytes treated with RAd-IGF-I (Figure 6). IL6 and IL-1β mRNA levels 72 h after incubation with RAd-IGF-I were not significantly different from those in uninfected astrocytes (Figure 6). TNF-α mRNA levels were increased 72 h after incubation with RAd-IGF-I. TLR4 mRNA levels were significantly decreased 24 h after incubation of the cultures with RAd-IGF-I, compared to astrocytes treated with RAd-TK/GFP (Figure 6).

### RAd-IGF-I exerts an anti-inflammatory action on LPS-treated astrocytes

**Figure 7:**
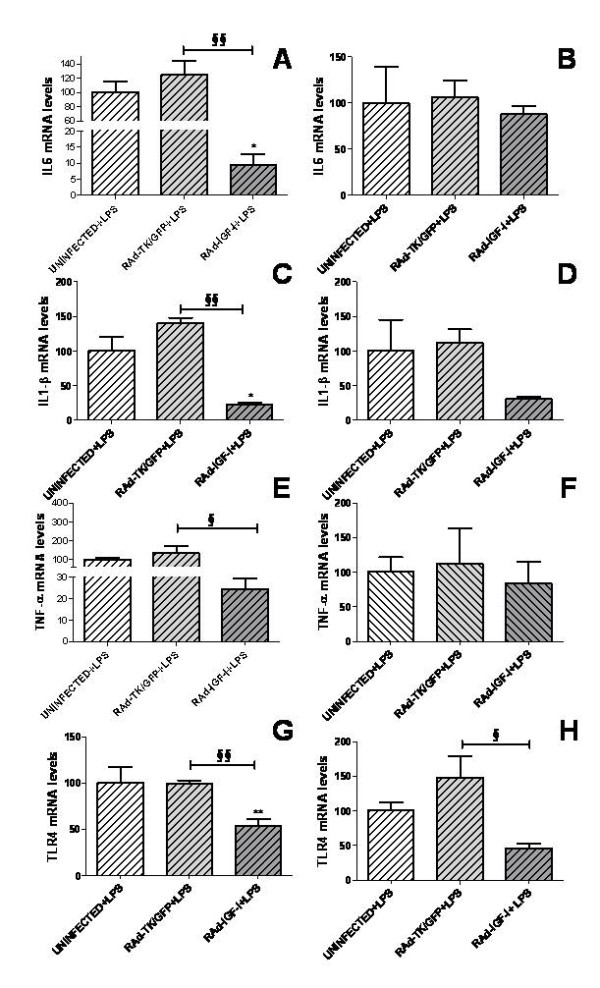
**RAd-IGF-I decreases the expression of proinflammatory molecules in LPS-stimulated astrocytes**. mRNA levels of IL6 (A,B), IL-1β (C,D), TNF-α (E,F) and TLR4 (G,H) in astrocytes 24 h (A,C,E,G) or 72 h (B,D,F,H) after the addition of RAd-TK/GFP or RAd-IGF-I and treated for the last 5 h with LPS. Data are presented as mean ± SEM and are expressed as percentage of control (uninfected cells treated with LPS) values. *,**, Significant differences (* P < 0.05; **P < 0.01) in comparison to the values of uninfected cells. ^**§**^, ^**§§**^, Significant differences (^**§**^P < 0.05; ^**§§**^P < 0.01) between RAd-TK/GFP and RAd-IGF-I.

After LPS treatment, astrocytes incubated with RAd-IGF-I showed a significant decrease in mRNA levels for IL6, IL-1β and TNF-α compared to astrocytes incubated with RAd-TK/GFP (Figure 7). In addition, IL6 and IL-1β mRNA levels were significantly decreased in cultures incubated with RAd-IGF-I compared to uninfected cultures. In LPS-treated astrocytes, TLR4 mRNA levels were significantly decreased in cultures incubated for 24 h or 72 h with RAd-IGF-I compared to astrocytes treated with RAd-TK/GFP. In addition, TLR4 mRNA levels were significantly decreased in astrocytes incubated with RAd-IGF-I for 24 h compared to uninfected astrocytes.

### Role of IGF-I receptor in the effects of RAd-IGF-I

**Figure 8:**
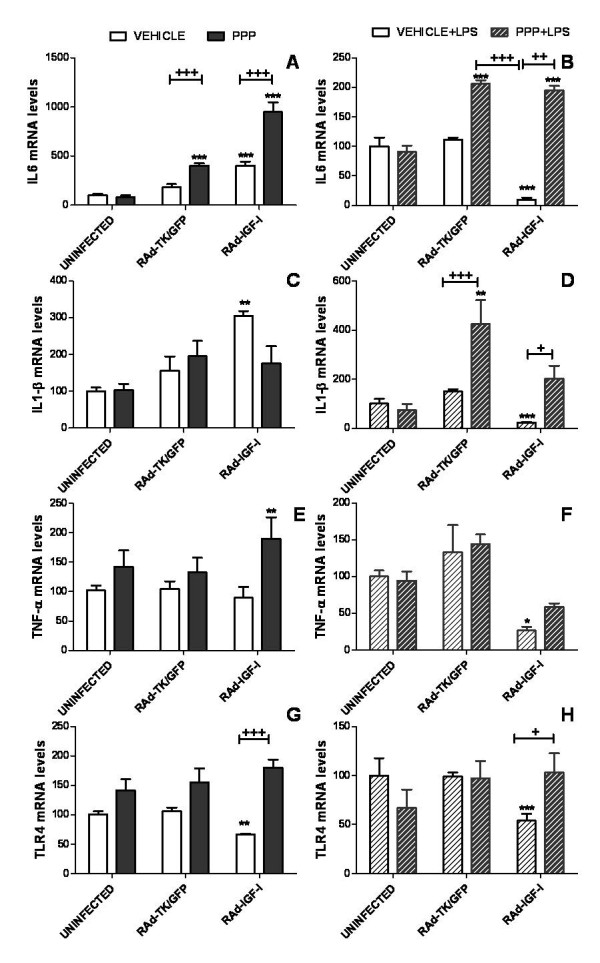
**Effects of an IGF-I receptor antagonist on uninfected astrocytes and on astrocytes incubated with the adenoviral vectors**. mRNA levels of IL6 (A,B), IL-1β (C,D), TNF-α (E,F) and TLR4 (G,H) in astrocytes 24 h after the addition of RAd-TK/GFP or RAd-IGF-I and treated for the last 5 h with the IGF-I receptor tyrosine kinase inhibitor PPP or its vehicle in the absence (A,C,E,G) or the presence of LPS (B,D,F,H). Data are presented as mean ± SEM and are expressed as percentage of control (uninfected cells treated with the vehicle for PPP) values. *,**,***, Significant differences (* P < 0.05; **P < 0.01; ***P < 0.001) in comparison to control values. +,++,+++, Significant differences between PPP and its vehicle.

To determine whether the effects of RAd-IGF-I on IL6, IL-1β, TNF-α and TLR4 mRNA levels are mediated by IGF-I receptor, astrocyte cultures were treated with the IGF-I receptor antagonist cyclolignan picropodophyllin (PPP). As shown in figure 8, PPP did not block the effect of RAd-IGF-I on IL6 mRNA levels under basal, un-challenged conditions (Figure 8A). In fact, PPP treatment increased IL6 mRNA abundance in cells infected with the Rad-IGF-I construct or the control construct. In contrast, PPP blocked the increase in IL-1β mRNA levels induced by RAd-IGF-I under basal conditions (Figure 8C). PPP also resulted in an increase in TNF-α and TLR4 mRNA levels in cultures treated with RAd-IGF-I (Figure 8 E,G), preventing the decrease in TLR4 mRNA levels induced by RAd-IGF-I under basal conditions (Figure 8G).

In astrocytes treated with LPS, the effects of RAd-IGF-I on IL6, IL-1β, TNF-α and TLR4 mRNA levels were reverted by incubating the cultures for 5 h with PPP (Figure 8B,D,F,H). In addition PPP increased mRNA levels of IL6 and IL-1β in LPS-treated astrocytes incubated with RAd-TK/GFP or RAd-IGF-I (Figure 8B,D).

### RAd-IGF-I prevents LPS-induced nuclear translocation of the NFκB p65 subunit

**Figure 9:**
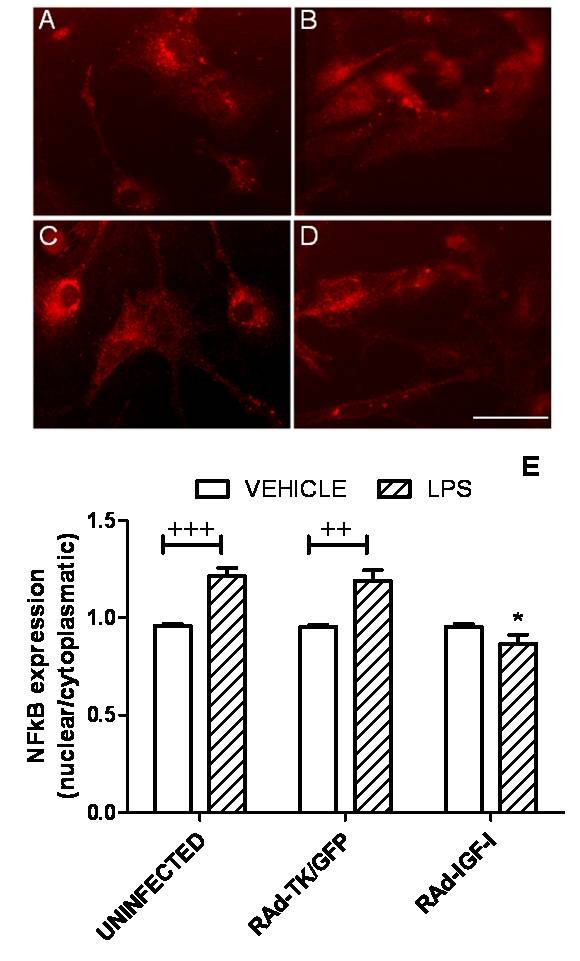
**RAd-IGF-I prevents LPS-induced nuclear translocation of the NFκB p65 subunit**. Astrocyte cultures were treated for 24 h with RAd-IGF-I and for 1 h with LPS, and the level of p65 immunoreactivity in nucleus and cytoplasm was determined. A-D, Representative examples of p65 immunostaining in astrocytes incubated with RAd-TK/GFP (A), RAd-TK/GFP + LPS (B), RAd-IGF-I (C) and RAd-IGF-I + LPS (D), Scale bar, 40 μm. E, Results of densitometric analysis of p65 immunoreactivity expressed as a ratio between immunoreactivity in the cell nucleus and the cell cytoplasm. Data are presented as mean ± SEM and are expressed as percentage of control (uninfected cells treated with vehicle) values. ^++^,^+++ ^Significant differences (^++^P < 0.01, ^+++^P < 0.001) between LPS and its vehicle. *, Significant difference (*P < 0.05) versus uninfected + LPS and versus RAd-TK/GFP + LPS values.

Since activation of TLR4 results in nuclear translocation of the NFκB p65 subunit, we assessed whether RAd-IGF-I affects p65 subcellular distribution in astrocytes. The incubation of astrocytes with RAd-TK/GFP and LPS resulted in a significant increase in p65 immunoreactivity in the cell nucleus compared to astrocytes incubated with RAd-TK/GFP alone. In contrast, p65 nuclear immunoreactivity was not increased by LPS in astrocytes incubated with RAd-IGF-I (Figure 9). Therefore, RAd-IGF-I prevented LPS-induced p65 nuclear translocation.

## Discussion

The findings of the present study indicate that IGF-I increases the expression of IL6, IL-1β and TNF-α in cultured astrocytes under basal conditions. In contrast, IGF-I decreases expression of IL6 and IL-1β in cultured astrocytes submitted to a proinflammatory challenge by treatment with LPS. Under basal conditions, the effects of IGF-I on expression of IL6, IL-1β and TNF-α were dependent on IGF-I dose, promoting these cytokine expressions at the lower dose. When challenged with LPS, however, IGF-I at both dosages exerted similar inhibitory effects on the expression of IL6 and IL-1β.

The increases in IL6, IL-1β and TNF-α mRNA levels detected in astrocytes after treatment with IGF-I under basal conditions does not necessarily imply a proinflammatory action of IGF-I. Cytokines such as IL6, IL-1β and TNF-α have different physiological functions, including regulation of neuronal development, ionic homeostasis, neuropeptide release and synaptic plasticity [31-34]. Therefore, the observed increases in IL6, IL-1β and TNF-α levels under basal conditions may represent a physiological action of IGF-I. In addition, IGF-I reduced expression of TLR4 in both the presence and absence of LPS. TLR4 is a member of the IL-1receptor/TLR superfamily that is expressed by astrocytes and that is required for an LPS-induced inflammatory response [35-38]. Therefore the observed decrease in TLR4 expression by IGF-I under basal conditions and after LPS proinflammatory challenge may contribute to a reduced capacity of astrocytes to be activated by proinflammatory molecules such as LPS. Indeed, IGF-I reduced the effects of LPS on mRNA levels of IL6, and IL-1β in astrocytes, in agreement with previous findings showing that IGF-I down-regulates cytokine expression induced by LPS in adult mouse brain [39].

Having established that exogenous IGF-I regulates the expressions of IL6, IL-1β, TNF-α and TLR4 in astrocytes, we then assessed whether an increase in endogenous production of IGF-I by astrocytes might also affect expression of these molecules. The findings of the present study indicate that a recombinant adenoviral vector harboring the gene for rat IGF-I (RAd-IGF-I) is able to increase the production of IGF-I in astrocytes, without affecting IGFBP2 or IGF-I receptor levels. Under basal conditions, RAd-IGF-I resulted in an effect similar to that of IGF-I, increasing the expression of IL6 and IL-1β and decreasing the expression of TLR4. Adenoviral vector infection per se also had an effect, since mRNA levels of IL6 and IL-1β were increased in astrocytes 72 h after incubation with control vector, RAd-TK/GFP. In addition, TNF-α mRNA levels were increased in astrocytes incubated for 72 h with RAd-IGF-I. However, no increase for IL6 or IL-1β was detected at 72 h in astrocytes infected with RAd-IGF-I, suggesting that IGF-I is compensating for some of the proinflammatory actions of the adenoviral vector. Indeed, RAd-IGF-I exerted a clear anti-inflammatory action on LPS-treated astrocytes, decreasing the mRNA levels of IL6, IL-1β, TNF-α and TLR4. Therefore, RAd-IGF-I imitated the effects of IGF-I on LPS-stimulated astrocytes. These findings demonstrate efficacy of IGF-I gene therapy in reducing the response of astrocytes to an inflammatory challenge. However, as was observed for exogenous IGF-I, the anti-inflammatory action of IGF-I gene therapy was transient, since RAd-IGF-I did not significantly affect levels of IL6, IL-1β, or TNF-α in cultures incubated with the viral vector for 72 h.

Our data suggest that RAd-IGF-I, like IGF-I, reduces the effects of LPS on astrocytes by decreasing the expression of TLR4. Activation of TLR4 results in translocation of the NFκB p65 subunit to the cell nucleus and consequent activation of NFκB-mediated transcription of proinflammatory cytokines and chemokines [40-44]. In agreement with the effect of RAd-IGF-I on TLR4 expression, and with the results of a previous study on the effect of IGF-I on astrocytes [18], IGF-I gene therapy prevented LPS-induced translocation of the NFκB p65 subunit to the cell nucleus. This suggests that RAd-IGF-I exerts, at least in part, its anti-inflammatory action by downregulation of TLR4 and subsequent inhibition of NFκB activity.

The anti-inflammatory action of RAd-IGF-I in LPS-stimulated astrocytes may be mediated by IGF-I receptor activation, since it was prevented by incubation of astrocytes with cyclolignan picropodophyllin (PPP), an inhibitor of the IGF-I receptor tyrosine phosphorylation [45-47]. However, PPP treatment per se increased IL6 mRNA levels in cells infected with Rad-IGF-I construct or control construct under basal conditions, and increased mRNA levels of IL6 and IL-1β in LPS-treated astrocytes incubated with RAd-TK/GFP or RAd-IGF-I. These actions of PPP cannot be explained simply by IGF-I receptor inhibition, suggesting that PPP may have other effects under the experimental conditions used.

## Conclusion

The results of the present study indicate that IGF-I and IGF-I gene therapy are able to reduce inflammatory reactions in glial cells exposed to LPS. This action of IGF-I and IGF-I gene therapy may be relevant for control of reactive gliosis under chronic neurodegenerative conditions, where the glial inflammatory response is thought to have a negative impact on neuronal function and survival.

## List of abbreviations used

BSA: bovine serum albumin; ANOVA: analysis of variance; DMEM: Dulbecco's modified Eagle medium; EDTA: ethylenediaminetetraacetic acid; GAPDH: glyceraldehyde-3-phosphate dehydrogenase; GFP: green fluorescent protein; HEK293: human embryo kidney 293 cells; IGF-I: insulin-like growth factor-I; IL-1β: interleukin 1β; IL6: interleukin 6; LPS: lipopolysaccharide; mCMV: mouse cytomegalovirus; NFκB: nuclear factor κB; PPP: picropodophyllin; RAd: recombinant adenovirus; TK: thymidine kinase; TLR4: toll-like receptor 4; TNF-α: tumor necrosis factor-α.

## Competing interests

The authors declare that they have no competing interests.

## Authors' contributions

MJB carried out the design and the experimental work, CBH constructed the adenoviral vectors used, RGG contributed to vector design and manuscript preparation and MJB and LMGS contributed to the analysis of the data and manuscript preparation. All authors read and approved the final manuscript.

## References

[B1] Tuppo EE Arias HR The role of inflammation in Alzheimer's disease The International Journal of Biochemistry & Cell Biology 2005 37 289 305 10.1016/j.biocel.2004.07.009 15474976

[B2] Holley JE Gveric D Newcombe J Cuzner ML Gutowski NJ Astrocyte characterization in the multiple sclerosis glial scar Neuropathology and Applied Neurobiology 2003 29 434 444 10.1046/j.1365-2990.2003.00491.x 14507335

[B3] Volterra A Meldolesi J Astrocytes, from brain glue to communication elements: the revolution continues Nature Review Neurosciences 2005 6 626 640 10.1038/nrn1722 16025096

[B4] Farina C Aloisi F Meinl E Astrocytes are active players in cerebral innate immunity Trends in Immunology 2007 28 138 145 10.1016/j.it.2007.01.005 17276138

[B5] Cerciat M Unkila M Garcia-Segura LM Arevalo MA Selective estrogen receptor modulators decrease the production of interleukin-6 and interferon-γ-inducible protein-10 by astrocytes exposed to inflammatory challenge in vitro Glia 2010 58 93 102 10.1002/glia.20904 19533603

[B6] Fisher L Samuelsson M Jiang Y Ramberg V Figueroa R Hallberg E Targeting cytokine expression in glial cells by cellular delivery of an NF-κB decoy Journal of Molecular Neuroscience 2007 31 209 219 17726227 10.1385/jmn:31:03:209

[B7] Kipp M Karakaya S Pawlak J raujo-Wright G Arnold S Beyer C Estrogen and the development and protection of nigrostriatal dopaminergic neurons: Concerted action of a multitude of signals, protective molecules, and growth factors Frontiers in Neuroendocrinology 2006 27 376 390 10.1016/j.yfrne.2006.07.001 16949139

[B8] Buchanan MM Hutchinson M Watkins LR Yin H Toll-like receptor 4 in CNS pathologies Journal of Neurochemistry 2010 114 13 27 20402965 10.1111/j.1471-4159.2010.06736.x PMC2909662

[B9] Guan J Skinner SJM Beilharz EJ Hua KM Hodgkinson S Gluckman PD The movement of IGF-I into the brain parenchyma after hypoxic-ischaemic injury NeuroReport 1996 7 632 636 10.1097/00001756-199601310-00061 8730846

[B10] Carro E Torres-Aleman I Serum insulin-like growth factor I in brain function The Keio Journal of Medicine 2006 55 59 63 10.2302/kjm.55.59 16830417

[B11] Aleman A Torres-Aleman I Circulating insulin-like growth factor I and cognitive function: Neuromodulation throughout the lifespan Progress in Neurobiology 2009 89 256 265 10.1016/j.pneurobio.2009.07.008 19665513

[B12] Torres-Aleman I Insulin-like growth factors as mediators of functional plasticity in the adult brain Hormone Metabolism Research 1999 31 114 119 10.1055/s-2007-978707 10226790

[B13] Åberg ND Brywe KG Isgaard J Brywe KG Aspects of growth hormone and insulin-like growth factor-I related to neuroprotection, regeneration, and functional plasticity in the adult brain TheScientificWorldJOURNAL 2006 6 53 80 16432628 10.1100/tsw.2006.22 PMC5917186

[B14] Fernández S García-García M Torres-Alemán I Modulation by insulin-like growth factor I of the phosphatase PTEN in astrocytes Biochimica et Biophysica Acta (BBA) - Molecular Cell Research 2008 1783 803 812 10.1016/j.bbamcr.2007.10.020 18062928

[B15] Carro E Trejo JL Busiguina S Torres-Aleman I Circulating insulin-like growth factor I mediates the protective effects of physical exercise against brain Insults of different etiology and anatomy Journal of Neuroscience 2001 21 5678 5684 11466439 10.1523/JNEUROSCI.21-15-05678.2001 PMC6762673

[B16] Carro E Trejo J Nuñez A Torres-Aleman I Brain repair and neuroprotection by serum insulin-like growth factor I Molecular Neurobiology 2003 27 153 162 10.1385/MN:27:2:153 12777685

[B17] Venters HD Dantzer R Kelley KW A new concept in neurodegeneration: TNF[alpha] is a silencer of survival signals Trends in Neurosciences 2000 23 175 180 10.1016/S0166-2236(99)01533-7 10717677

[B18] Pons S Torres-Aleman I Insulin-like growth factor-I stimulates dephosphorylation of IκB through the serine phosphatase calcineurin (protein phosphatase 2B) Journal of Biological Chemistry 2000 275 38620 38625 10.1074/jbc.M004531200 10973957

[B19] Fernandez AM Garcia-Estrada J Garcia-Segura LM Torres-Aleman I Insulin-like growth factor I modulates c-fos induction and astrocytosis in response to neurotoxic insult Neuroscience 1996 76 117 122 10.1016/S0306-4522(96)00395-8 8971764

[B20] Garcia-Estrada J Garcia-Segura LM Torres-Aleman I Expression of insulin-like growth factor I by astrocytes in response to injury Brain Research 1992 592 343 347 10.1016/0006-8993(92)91695-B 1280521

[B21] Ye P Popken GJ Kemper A McCarthy K Popko B D'Ercole AJ Astrocyte-specific overexpression of insulin-like growth factor-I promotes brain overgrowth and glial fibrillary acidic protein expression J Neuroscience Research 2004 78 472 484 10.1002/jnr.20288 15468174

[B22] O'Donnell SL Frederick TJ Krady JK Vannucci SJ Wood TL IGF-I and microglia/macrophage proliferation in the ischemic mouse brain Glia 2002 39 85 97 12112378 10.1002/glia.10081

[B23] Walter HJ Berry M Hill DJ Logan A spatial and temporal changes in the insulin-like growth factor (IGF) axis indicate autocrine/paracrine actions of IGF-I within wounds of the rat brain Endocrinology 1997 138 3024 3034 10.1210/en.138.7.3024 9202248

[B24] Hereñú CB Cristina C Rimoldi OJ Becu-Villalobos D Cambiaggi V Portiansky EL Restorative effect of insulin-like growth factor-I gene therapy in the hypothalamus of senile rats with dopaminergic dysfunction Gene Theraphy 2006 14 237 245 10.1038/sj.gt.3302870 16988717

[B25] Hereñú CB Sonntag WE Morel GR Portiansky EL Goya RG The ependymal route for insulin-like growth factor-1 gene therapy in the brain Neuroscience 2009 163 442 447 19531373 10.1016/j.neuroscience.2009.06.024 PMC2740751

[B26] Hitt M Bett AJ Prevec L Graham FL JE Celis Construction and propagation of human adenovirus vectors. 1500-1512 1998 Cell Biology: A Laboratory Handbook. Academic Press: San Diego, CA

[B27] Daughaday WH Rotwein P Insulin-like growth factors I and II. Peptide, messenger ribonucleic acid and gene structures, serum, and tissue concentrations Endocrine Reviews 1989 10 68 91 10.1210/edrv-10-1-68 2666112

[B28] Paquin A Jaalouk DE Galipeau J Retrovector encoding a green fluorescent protein - herpes simplex virus thymidine kinase fusion protein serves as a versatile suicide/reporter for cell and gene therapy applications Human Gene Therapy 2004 12 13 23 10.1089/104303401450924 11177538

[B29] Rubio N Gonzalez-Tirante M Arevalo MA Aranguez I Over-expression of GTP-binding proteins and GTPase activity in mouse astrocyte membranes in response to Theiler's murine encephalomyelitis virus infection Journal of Neurochemistry 2008 104 100 112 17995937 10.1111/j.1471-4159.2007.05020.x

[B30] Pistritto G Franzese O Pozzoli G Mancuso C Tringali G Preziosi P Bacterial lipopolysaccharide increases prostaglandin production by rat astrocytes via inducible cyclo-oxygenase: Evidence for the involvement of nuclear factor [kappa]B Biochemical and Biophysical Research Communications 1999 263 570 574 10.1006/bbrc.1999.1413 10491333

[B31] Raber J Sorg O Horn TF Yu N Koob GF Campbell IL Bloom FE Inflammatory cytokines: putative regulators of neuronal and neuro-endocrine function Brain Research Brain Research Reviews 1998 26 320 326 10.1016/S0165-0173(97)00041-6 9651548

[B32] Albensi BC Mattson MP Evidence for the involvement of TNF and NF-κB in hippocampal synaptic plasticity Synapse 2000 35 151 159 10.1002/(SICI)1098-2396(200002)35:2<151::AID-SYN8>3.0.CO;2-P 10611641

[B33] Skoff AM Zhao C Adler JE Interleukin-1alpha regulates substance P expression and release in adult sensory neurons Experimental Neurology 2009 217 395 400 10.1016/j.expneurol.2009.03.022 19341730

[B34] Park KM Bowers WJ Tumor necrosis factor-alpha mediated signaling in neuronal homeostasis and dysfunction Cellular Signalling 2010 22 977 983 10.1016/j.cellsig.2010.01.010 20096353 PMC2860549

[B35] Blanco AM Valles SL Pascual M Guerri C Involvement of TLR4/type I IL-1 receptor signaling in the induction of inflammatory mediators and cell death induced by ethanol in cultured astrocytes Journal of Immunology 2005 175 6893 6899 10.4049/jimmunol.175.10.6893 16272348

[B36] Konat GW Krasowska-Zoladek A Kraszpulski M Statins enhance toll-like receptor 4-mediated cytokine gene expression in astrocytes: Implication of Rho proteins in negative feedback regulation Journal of Neuroscience Research 2008 86 603 609 10.1002/jnr.21509 17896797

[B37] Krasowska-Zoladek A Banaszewska M Kraszpulski M Konat GW Kinetics of inflammatory response of astrocytes induced by TLR 3 and TLR4 ligation Journal of Neuroscience Research 2007 85 205 212 10.1002/jnr.21088 17061254

[B38] Liao CK Wang SM Chen YL Wang HS Wu JC Lipopolysaccharide-induced inhibition of connexin43 gap junction communication in astrocytes is mediated by downregulation of caveolin-3 The International Journal of Biochemistry & Cell Biology 2010 42 762 770 10.1016/j.biocel.2010.01.016 20093193

[B39] Park SE Dantzer R Kelley KW McCusker RH Central administration of insulin-like growth factor-I decreases depressive-like behavior and brain cytokine expression in mice Journal of Neuroinflammation 2011 8 12 10.1186/1742-2094-8-12 21306618 PMC3045937

[B40] Arimilli S Johnson JB exander-Miller MA Parks GD TLR-4 and -6 agonists reverse apoptosis and promote maturation of simian virus 5-infected human dendritic cells through NFkB-dependent pathways Virology 2007 365 144 156 10.1016/j.virol.2007.02.035 17459446 PMC1949023

[B41] Földes G von Haehling S Okonko DO Jankowska EA Poole-Wilson PA Anker SD Fluvastatin reduces increased blood monocyte Toll-like receptor 4 expression in whole blood from patients with chronic heart failure International Journal of Cardiology 2008 124 80 85 17383753 10.1016/j.ijcard.2006.12.024

[B42] Wittebole X Castanares-Zapatero D Laterre PF Toll-like receptor 4 modulation as a strategy to treat sepsis Mediators of Inflammation 2010 2010 568396 10.1155/2010/568396 20396414 PMC2855078

[B43] Lin ST Wang Y Xue Y Feng DC Xu Y Xu LY Tetrandrine suppresses LPS-induced astrocyte activation via modulating IKKs-IκBα-NF-κB signaling pathway Molecular and Cellular Biochemistry 2008 315 41 49 10.1007/s11010-008-9787-4 18498042

[B44] Park C Lee S Cho IH Lee HK Kim D Choi SY TLR3-mediated signal induces proinflammatory cytokine and chemokine gene expression in astrocytes: Differential signaling mechanisms of TLR3-induced IP-10 and IL-8 gene expression Glia 2006 53 248 256 10.1002/glia.20278 16265667

[B45] Girnita A Girnita L Prete F Bartolazzi A Larsson O Axelson M Cyclolignans as inhibitors of the insulin-like growth factor-1 receptor and malignant cell growth Cancer Research 2004 64 236 242 10.1158/0008-5472.CAN-03-2522 14729630

[B46] Vasilcanu D Girnita A Girnita L Vasilcanu R Axelson M Larsson O The cyclolignan PPP induces activation loop-specific inhibition of tyrosine phosphorylation of the insulin-like growth factor-1 receptor. Link to the phosphatidyl inositol-3 kinase//Akt apoptotic pathway Oncogene 2004 23 7854 7862 10.1038/sj.onc.1208065 15334055

[B47] Stromberg T Ekman S Girnita L Dimberg LY Larsson O Axelson M IGF-1 receptor tyrosine kinase inhibition by the cyclolignan PPP induces G2/M-phase accumulation and apoptosis in multiple myeloma cells Blood 2006 107 669 678 10.1182/blood-2005-01-0306 16166596

